# A novel miR‐82 target PA2G4 and FHL3 checking myocardial ischemia

**DOI:** 10.1002/ctm2.179

**Published:** 2020-09-22

**Authors:** Ruixia Kang, Bing Li, Yingying Zhang, Qiong Liu, Jun Liu, Yifei Qi, Fei Teng, Huaiqian Xu, Zhenhua Liu, Xiaolong Zhu, Wangmin Qiao, Jinzhou Tian, Yimin Wang, Jingyi Nan, Jian Guo, Zhong Wang

**Affiliations:** ^1^ Institute of Basic Research in Clinical Medicine China Academy of Chinese Medical Sciences Beijing China; ^2^ Institute of Information on Traditional Chinese Medicine China Academy of Chinese Medical Sciences Beijing China; ^3^ Dongzhimen Hospital Beijing University of Chinese Medicine Beijing China; ^4^ Beijing Genomics Institute Shenzhen China; ^5^ Shananxi Buchang Pharmaceutical Co., Ltd. Xianyang China

Dear Editor,

The incidence and mortality of ischemic heart disease increase all over the world, producing immense health and economic burdens.^1^ MicroRNAs (miRNAs) regulate multiple biological processes.^2^ It has been identified thousands of distinct miRNAs in humans and model organisms. The expression of miRNAs in heart tissues has been studied.^3^ miRNAs can be potentially used as therapeutic targets and noninvasive clinical biomarkers.^4^

A novel miR‐82 was discovered in a multicenter, double‐blind, randomized, large‐scale, and placebo‐controlled clinical study. The efficacy of Danhong injection (DHI, a Chinese materia medica standardized product, which is widely used to treat coronary heart disease) in the treatment of chronic stable angina was evaluated (ClinicalTrials.gov, NCT01681316).^5^ The homologous sequence of miR‐82 was not found in other species by sequence alignment in databases.

The level of novel miR‐82 was decreased in the therapeutically effective patients (Figure 1A) and increased in the therapeutically ineffective patients (Figure 1B) in the DHI group. It was indicated that miR‐82 upregulation may be associated with the stable angina treatment of DHI and its downregulation may have a protective effect on myocardial ischemia. miR‐82 may be a new biomarker and therapeutic target for myocardial ischemia. The structure and sequence of miR‐82 were identified as follows (Figure 1C, completed by Shenzhen Huada Gene Technology Service Co., Ltd.). We experimentally confirmed the existence of miR‐82 in AC16 and H9C2 cells with qRT‐PCR and northern blot analysis (Figure 1D and E). The relative expression of miR‐82 from six types of rat tissues is shown in Figure 1F. These results revealed the existence of miR‐82 in diverse rat tissues. The level of miR‐82 in blood was the most abundant, followed by the heart, which was nearly equivalent in liver, lung, and kidney, and least in the spleen. These results suggested that miRNA‐82 was not human specific, which was also expressed in rats.

**FIGURE 1:**
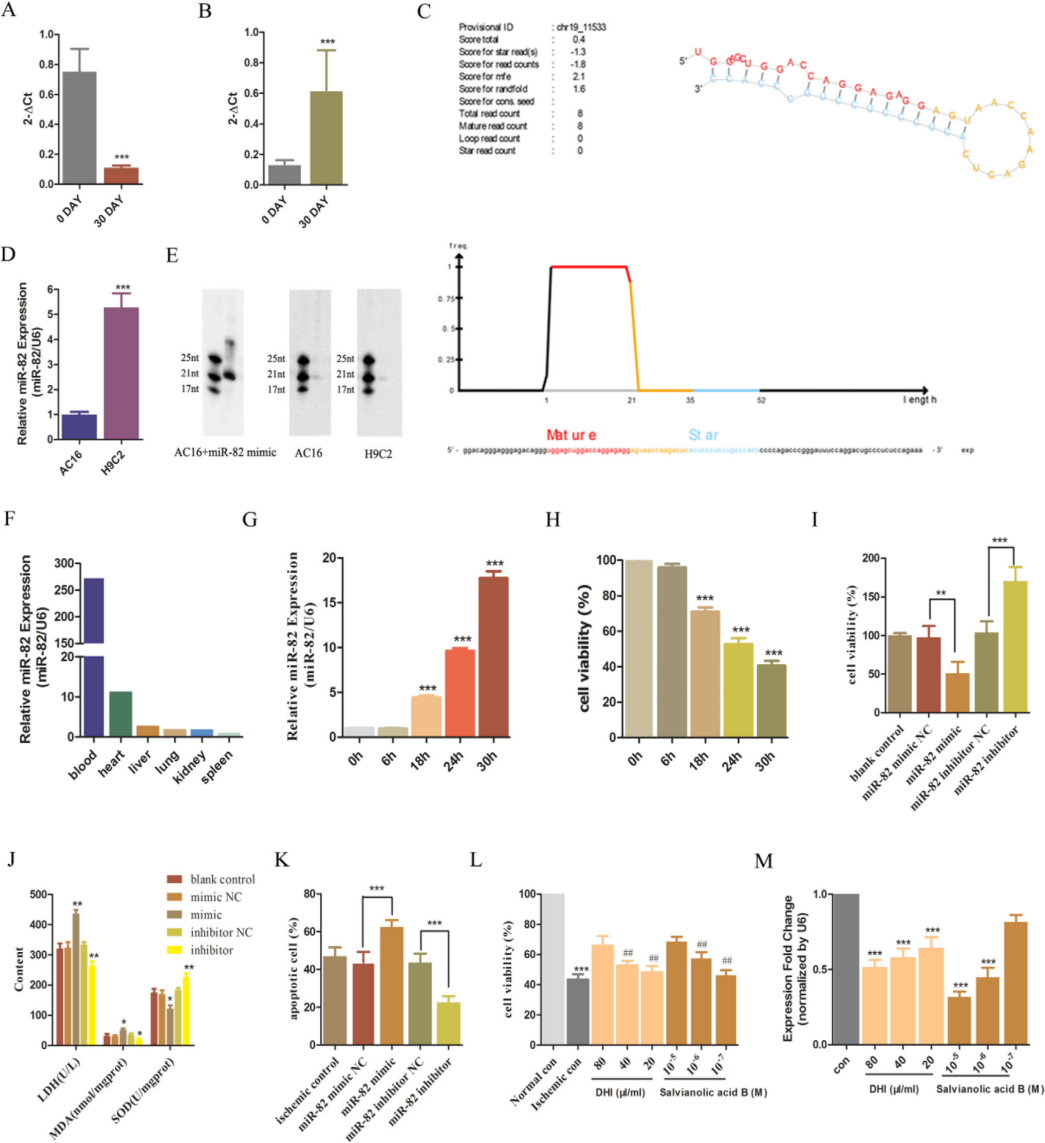
The discovery and effect of miR‐82 on ischemic AC16 cells. A, The relative expression of miR‐82 in therapeutically effective patients in the DHI group. ^***^ *P* < .001 compared with 0 day treated with DHI. B, The relative expression of miR‐82 in therapeutically ineffective patients in the DHI group. ^***^ *P* < .001 compared with 30 days treated with DHI. C, The sequence and secondary structure of miR‐82. D, The relative expression of miR‐82 in AC16 cells and H9C2 cells. E, Northern blot analysis of miRNA‐82 expression. F, The relative expression of miR‐82 from different organs in Wistar rats. G, Effect of miR‐82 mimic/ inhibitor on proliferation for ischemic AC16. H, Effect of miR‐82 mimic/ inhibitor on the contents of LDH, MDA, and SOD after ischemic treatment. I, Effect of miR‐82 mimic/ inhibitor on apoptosis for ischemic AC16. ^*^ *P* < .05, ^**^ *P* < .01, ^****^ *P* < .001 compared with the negative control. N = 5. J, Effect of DHI or salvianolic acid B on ischemic AC16. ^***^ *P* < .001 compared with the normal control. ^###^ *P* < .001, ^##^ *P* < .01, ^#^ *P* < .05 compared with the ischemic control. N = 5. K, miR‐82 expression for ischemic AC 16 in the absence or presence of DHI or salvianolic acid B. N = 5. ***P<.001, **P<.01, *P <.05 compared with control

The miR‐82 expression was upregulated in ischemic AC16 in a time‐dependent manner (Figure 1G). As AC16 exposed to ischemia for 24 hours resulted in approximately 50% cell death (Figure 1H) and 9.4‐fold miR‐82 upregulation (Figure 1G), AC16 cells were treated in tri‐gas incubator for 24 hours in all subsequent ischemia‐related experiments. We transfected miR‐82 mimic and miR‐82 inhibitor into AC16 cells and explored the effect of miR‐82 in myocardial ischemia in vitro. The transfection with miR‐82 mimic decreased the cell viability (Figure 1I), increased lactate dehydrogenase (LDH), and intracellular Malondialdehyde (MDA) content in AC16 cell supernatant and decreased intracellular Superoxide dismutase (SOD) content (Figure 1J), and increased the apoptosis rate (Figure 1K). Although inhibition of miR‐82 by transfection with miR‐82 inhibitor led to the opposite results, which had protective effect on ischemic AC16 cells (Figure 2C‐F). These results indicated that the inhibition of miR‐82 has protective effects on ischemic cardiomyocytes. Incubation of ischemic AC16 cells with DHI (80, 40, and 20 μL/mL) or salvianolic acid B (10^−5^, 10^−6^, and 10^−7^ M) caused a dose‐dependent increase (Figure 1G) in the viability and decrease of the miR‐82 expression (Figure 1H). It was indicated that the miR‐82 downregulation may be involved in the stable angina treatment of DHI.

**FIGURE 2:**
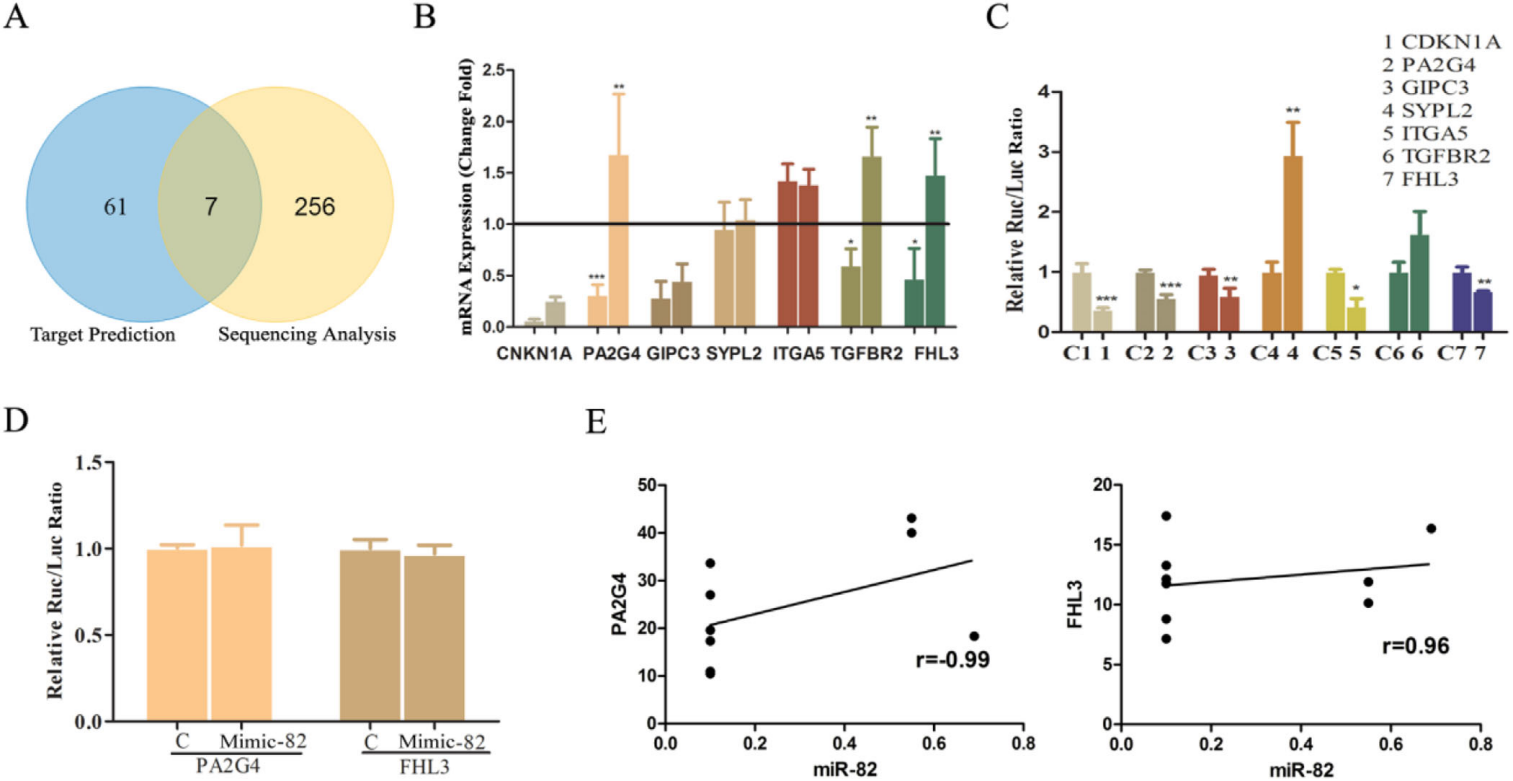
Identification of two targets of miR‐82 in ischemic process. A, The results of miR‐82 targets prediction with bioinformatic tools (left circle) and inhibited targets by miR‐82 performed by whole‐genome sequencing (right circle). B, The effect of miR‐82 to seven predicted target genes expression by qRT‐PCR. N = 5. C, Luciferase reporter assay with the pmiR‐targetgene‐3′‐UTR‐wt. N = 3. D, Luciferase reporter assay with pmiR‐mutant‐3′‐UTR‐wt. N = 3. E, Pearson's coefficients of miR‐82 and PA2G4 (left, Pearson *r* = −0.99, N = 8) or miR‐82 and FHL3 (right, Pearson *r* = 0.96, N = 9). ^*^ *P* < .05, ^**^ *P* < .01, ^***^ *P* < .001 compared with control

In order to identify the target genes that may interact with miR‐82, miRB, Targetscan, RNA22, and miRanda, four target prediction software were used. A total of 61 target genes were predicted by the four software. Whole‐genome sequencing analysis with Illumina HiSeqTM 2000 indicated that elevated expression of miR‐82 caused a total of 316 genes with significant expression differences, of which 256 were significantly downregulated. As shown in Figure 2A, the predicted 61 target genes shared seven overlapping genes with the 256 downregulated genes, CDKN1A, PA2G4, GIPC3, SYPL2, ITGA5, TGFBR2, and FHL3. The complementary sequences between miR‐82 and its putative sites within the 3′UTRs of seven overlapping genes, predicted with computational and bioinformatics‐based approach using TargetScan, are shown in Table S1. To confirm the targets genes of miR‐82, we performed qRT‐PCR and the luciferase binding assay. We detected the expression of the seven predicted target genes using qRT‐PCR. The results are shown in Figure 2B. The transfection with miR‐82 mimic could significantly inhibit the mRNA expression of PA2G4, TGFBR2, and FHL3 compared with the control group, and the transfection with miR‐82 inhibitor resulted in an increase in the expression of these three mRNAs. These results indicated that miR‐82 could regulate the expression of PA2G4, TGFBR2, and FHL3 mRNA in AC16 cells, suggesting that PA2G4, TGFBR2, and FHL3 may be target genes of miR‐82. Furthermore, we performed the luciferase reporter assay. As shown in Figure 2C, miR‐82 mimic caused a significant decrease in fluorescence of CDKN1A, PA2G4, GIPC3, ITGA5, and FHL3 wild‐type reporter vectors 64%, 46%, 40%, 53%, and 31%, respectively. Combined with the results of Figure 2B, we speculated that PA2G4 and FHL3 may be the direct targets of miR‐82. Next, we constructed mutant vectors of PA2G4 and FHL3, respectively, for confirmation. As shown in Figure 2D, miR‐82 mimic did not cause significant changes in fluorescence of PA2G4 and FHL3 mutant reporter vectors. These results indicated that PA2G4 and FHL3 are the direct target genes of miR‐82.

In the analysis of the correlation between miR‐82 and PA2G4 or FHL3 in clinical patients, there was a high negative correlation between miR‐82 and PA2G4, which was consistent with the results of target genes validation. However, miR‐82 was positively correlated with FHL3 (Figure 2E). The final conclusion may need to be validated in more clinical samples.

Taken together, we discovered a novel miR‐82 in a clinical trail and confirmed its expression. Our results provide the first evidence that the novel miR‐82 was not only expressed in human, but also in rats. miR‐82 was upregulated in ischemic AC16 cells and its downregulation had beneficial effect on ischemic AC16 cells. PA2G4 and FHL3 are the direct target genes of miR‐82. miR‐82 may be a druggable target for myocardial ischemia.

## CONFLICT OF INTEREST

The authors declare no conflict of interest.

## AUTHOR CONTRIBUTIONS

Zhong Wang designed this study. Ruixia Kang designed and performed this study and wrote the paper. Bing Li, Yingying Zhang, Fei Teng, and Qiong Liu analyzed the data. Qiong Liu and Yifei Qi were associated with the cell culture. Huaiqian Xu, Zhenhua Liu, Xiaolong Zhu, and Wangmin Qiao identified the structure and sequence of miR‐82. Jinzhou Tian, Yimin Wang, Jingyi Nan, and Jian Guo were associated with the designed. All authors approved the final manuscript.

## Supporting information

SUPPORTING INFORMATION
