# Supplementary material for: A novel miR‐82 target PA2G4 and FHL3 checking myocardial ischemia
Source: Clin Transl Med. 2020 Sep 22;10(5):e179. doi: 10.1002/ctm2.179 (PMC7508459; doi:10.1002/ctm2.179)
Supplement: Supplementary file 1 — SUPPORTING INFORMATION [file CTM2-10-e179-s001.docx]

**Supplementary**

Table 1. The complementary sequences between miR-82 and its putative sites within the 3’UTRs of selected genes, predicted with computational and bioinformatics-based approach using TargetScan. In the alignment, “ ׀ ” refers to perfect complementary between bases, “:” represents a G:U wobble pair.

| **miRNA** | **mRNA** | **Position** | **Structure** | **Loop Score** | **Energy**  **(kCal/Mol)** |
| --- | --- | --- | --- | --- | --- |
| novel_  mir_82 | CDKN1A | 1951:1968 | Query: 3' ggaGAGGACCAGGTCGAGGt 5'  \| \|\|\| \|\|\|\|\|\|\|\|\|  Ref: 5' aaaCACCT--TCCAGCTCCt 3' | 154.0 | -21.66 |
| novel_  mir_82 | CDKN1A | 1455:1474 | Query: 3' ggagaggaccaGGTCGAGGt 5'  :\|\|\|\|\|\|\|  Ref: 5' tcagtaccctcTCAGCTCCa 3' | 141.0 | -21.55 |
| novel_  mir_82 | TGFBR2 | 1499:1517 | Query: 3' ggagagGACCAGGTCGAGGt 5'  \|\|\|\| \|\|\|\|\|\|\|  Ref: 5' cgcaagCTGG-GCAGCTCCc 3' | 148.0 | -21.02 |
| novel_  mir_82 | TGFBR2 | 1126:1145 | Query: 3' ggaGAGGACCAGGTCGAGGt 5'  \|\|\|: :\|\|\|\|\|\|\|  Ref: 5' ccgCTCTGACATCAGCTCCa 3' | 145.0 | -23.78 |
| novel_  mir_82 | SYPL2 | 654:673 | Query: 3' ggagaggacCAGGTCGAGGt 5'  \|\|\|\|\|\|\|\|\|\|  Ref: 5' cgatgaagaGTCCAGCTCCa 3' | 155.0 | -23.21 |
| novel_  mir_82 | SYPL2 | 3011:3031 | Query: 3' ggagAGGAC-CAGGTCGAGGt 5'  \|::\|\| \| \|\|\|\|\|\|\|  Ref: 5' acagTTTTGCCTACAGCTCCa 3' | 147.0 | -20.20 |
| novel_  mir_82 | SYPL2 | 265:289 | Query: 3' ggAGA--GGACC---AGGTCGAGGt 5'  \|\|\| \|\| \|\| \| \|\|\|\|\|\|\|  Ref: 5' gcTCTGCCCCGGACCTGCAGCTCCc 3' | 144.0 | -22.00 |
| novel_  mir_82 | CDKN1A | 1896:1913 | Query: 3' ggaGAGGACCAGGTCGAGGt 5'  \| \|\|\| \|\|\|\|\|\|\|\|\|  Ref: 5' aaaCACCT--TCCAGCTCCt 3' | 154.0 | -21.66 |
| novel_  mir_82 | CDKN1A | 1896:1913 | Query: 3' ggagaggaccaGGTCGAGGt 5'  :\|\|\|\|\|\|\|  Ref: 5' tcagtaccctcTCAGCTCCa 3' | 141.0 | -21.55 |
| novel_  mir_82 | CDKN1A | 2061:2078 | Query: 3' ggaGAGGACCAGGTCGAGGt 5'  \| \|\|\| \|\|\|\|\|\|\|\|\|  Ref: 5' aaaCACCT--TCCAGCTCCt 3' | 154.0 | -21.66 |
| novel_  mir_82 | CDKN1A | 1565:1584 | Query: 3' ggagaggaccaGGTCGAGGt 5'  :\|\|\|\|\|\|\|  Ref: 5' tcagtaccctcTCAGCTCCa 3' | 141.0 | -21.55 |
| novel_  mir_82 | FHL3 | 912:932 | Query: 3' ggaGAGGACCAG-GTCGAGGt 5'  \|\|\|: \|\| \| \|\|\|\|\|\|\|  Ref: 5' cacCTCTGGGACTCAGCTCCc 3' | 156.0 | -25.20 |
| novel_  mir_82 | FHL3 | 1385:1405 | Query: 3' ggAG-AGGACCAGGTCGAGGt 5'  \|\| \|::\| \|:\|\|\|\|\|\|\|  Ref: 5' tcTCATTTTATTTCAGCTCCa 3' | 153.0 | -22.78 |
| novel_  mir_82 | CDKN1A | 2103:2120 | Query: 3' ggaGAGGACCAGGTCGAGGt 5'  \| \|\|\| \|\|\|\|\|\|\|\|\|  Ref: 5' aaaCACCT--TCCAGCTCCt 3' | 154.0 | -21.66 |
| novel_  mir_82 | CDKN1A | 1607:1626 | Query: 3' ggagaggaccaGGTCGAGGt 5'  :\|\|\|\|\|\|\|  Ref: 5' tcagtaccctcTCAGCTCCa 3' | 141.0 | -21.55 |
| novel_  mir_82 | ITGA5 | 3581:3599 | Query: 3' ggaGAGGACCAGGTCGAGGt 5'  :\|\|\|\| \|\|\|\|\|\|\|\|\|\|  Ref: 5' gacTTCCT-GTCCAGCTCCa 3' | 167.0 | -30.63 |
| novel_  mir_82 | ITGA5 | 3792:3812 | Query: 3' ggagaGGAC-CAGGTCGAGGt 5'  \|\| \| \| \|\|\|\|\|\|\|\|  Ref: 5' agggcCCAGAGCCCAGCTCCa 3' | 150.0 | -21.11 |
| novel_  mir_82 | ITGA5 | 2623:2644 | Query: 3' ggagaggaCCAG--GTCGAGGt 5'  \|\|\|\| \|\|\|\|\|\|\|  Ref: 5' ctctggaaGGTCAGCAGCTCCt 3' | 147.0 | -21.29 |
| novel_  mir_82 | ITGA5 | 2567:2586 | Query: 3' ggagaggaccaGGTCGAGGt 5'  \|\|\|\|\|\|\|\|  Ref: 5' caaccaaggccCCAGCTCCa 3' | 145.0 | -22.75 |
| novel_  mir_82 | ITGA5 | 1237:1254 | Query: 3' ggagaGGACCAGGTCGAGGt 5'  ::\|\|\| \|\|\|\|\|\|\|  Ref: 5' gccgaTTTGG--CAGCTCCt 3' | 144.0 | -20.71 |
| novel_  mir_82 | TGFBR2 | 1424:1442 | Query: 3' ggagagGACCAGGTCGAGGt 5'  \|\|\|\| \|\|\|\|\|\|\|  Ref: 5' cgcaagCTGG-GCAGCTCCc 3' | 148.0 | -21.02 |
| novel_  mir_82 | TGFBR2 | 1051:1070 | Query: 3' ggaGAGGACCAGGTCGAGGt 5'  \|\|\|: :\|\|\|\|\|\|\|  Ref: 5' ccgCTCTGACATCAGCTCCa 3' | 145.0 | -23.78 |
| novel_  mir_82 | FHL3 | 1088:1108 | Query: 3' ggaGAGGACCAG-GTCGAGGt 5'  \|\|\|: \|\| \| \|\|\|\|\|\|\|  Ref: 5' cacCTCTGGGACTCAGCTCCc 3' | 156.0 | -25.20 |
| novel_  mir_82 | FHL3 | 1561:1581 | Query: 3' ggAG-AGGACCAGGTCGAGGt 5'  \|\| \|::\| \|:\|\|\|\|\|\|\|  Ref: 5' tcTCATTTTATTTCAGCTCCa 3' | 153.0 | -22.78 |
| novel_  mir_82 | PA2G4 | 1756:1775 | Query: 3' ggagaggaccaGGTCGAGGt 5'  \|\|\|\|\|\|\|\|  Ref: 5' tccaacaacaaCCAGCTCCa 3' | 145.0 | -20.22 |
| novel_  mir_82 | CDKN1A | 1898:1915 | Query: 3' ggaGAGGACCAGGTCGAGGt 5'  \| \|\|\| \|\|\|\|\|\|\|\|\|  Ref: 5' aaaCACCT--TCCAGCTCCt 3' | 154.0 | -21.66 |
| novel_  mir_82 | CDKN1A | 1402:1421 | Query: 3' ggagaggaccaGGTCGAGGt 5'  :\|\|\|\|\|\|\|  Ref: 5' tcagtaccctcTCAGCTCCa 3' | 141.0 | -21.55 |
| novel_  mir_82 | GIPC3 | 2939:2958 | Query: 3' ggagagGACCAGGTCGAGGt 5'  \| \|\|\|\|\|\|\|\|\|\|\|  Ref: 5' cgacagCAGGTCCAGCTCCa 3' | 162.0 | -26.92 |
| novel_  mir_82 | GIPC3 | 2471:2490 | Query: 3' ggagaGGACCAGGTCGAGGt 5'  \|\| \|\|\|\|\|\|\|\|\|\|  Ref: 5' tgcagCCCAGTCCAGCTCCa 3' | 159.0 | -27.00 |
| novel_  mir_82 | GIPC3 | 1752:1770 | Query: 3' ggagAGGACCAGGTCGAGGt 5'  \|\|\| \|\| \|\|\|\|\|\|\|\|  Ref: 5' agagTCCAGG-CCAGCTCCg 3' | 158.0 | -25.09 |
| novel_  mir_82 | GIPC3 | 3023:3043 | Query: 3' ggaGAGGACCA-GGTCGAGGt 5'  :\|\|: \| \| \|\|\|\|\|\|\|\|  Ref: 5' aggTTCTAGATACCAGCTCCa 3' | 152.0 | -21.89 |
| novel_  mir_82 | GIPC3 | 2796:2814 | Query: 3' ggagAGGACCAGGTCGAGGt 5'  \|\|\| \| \|\|\|\|\|\|\|\|  Ref: 5' gaaaTCCCAG-CCAGCTCCa 3' | 150.0 | -22.38 |
| novel_  mir_82 | GIPC3 | 2220:2238 | Query: 3' ggagaGGACCAGGTCGAGGt 5'  :\|\|\|\| \|\|\|\|\|\|\|  Ref: 5' ggaacTCTGG-ACAGCTCCa 3' | 149.0 | -24.27 |
| novel_  mir_82 | GIPC3 | 3209:3228 | Query: 3' ggagaggaccaGGTCGAGGt 5'  \|\|\|\|\|\|\|\|  Ref: 5' cggagcccaacCCAGCTCCa 3' | 145.0 | -20.72 |
